# Characterization of Chromosome-Mediated *Bla*_OXA-894_ in *Shewanella xiamenensis* Isolated from Pig Wastewater

**DOI:** 10.3390/ijerph16193768

**Published:** 2019-10-08

**Authors:** Huiyun Zou, Ziyu Zhou, Huiyu Xia, Qian Zhao, Xuewen Li

**Affiliations:** Department of environment and health, School of Public Health, Shandong University, Jinan 250012, China; zouhuiyun@mail.sdu.edu.cn (H.Z.); zhouziyu199622@mail.sdu.edu.cn (Z.Z.); mosebyxhy@mail.sdu.edu.cn (H.X.); 201835834@mail.sdu.edu.cn (Q.Z.)

**Keywords:** *bla*_OXA-894_, *Shewanella xiamenensis*, genetic environment, pig wastewater

## Abstract

A new variant of the *bla*_OXA-546_ gene, namely *bla*_OXA-894_, was identified on the chromosome of *Shewanella xiamenensis* isolated from pig wastewater in rural China. OXA-894 differs from OXA-546 (A46V, I219*del*) and OXA-48 (T167I, I219*del*) with two amino acid substitutions, respectively. The isolate was resistant to ampicillin, aztreonam, imipenem, meropenem and fosfomycin. Carba NP test confirmed *S. xiamenensis* strain sx20 as a carbapenemase-producer. The *bla*_OXA-894_ gene was located between the gene encoding a LysR family transcriptional regulator and the C15 gene. Its gene environment was similar to other *S. xiamenensis* with chromosome-located *bla*_OXA-48_-like genes. The T24H and T94V amino acid substitutions of *LuxS* protein were predicted to be deleterious, which may affect the virulence phenotype. The occurrence and potential health risk of carbapenem-resistant *S. xiamenensis* in a water environment is of concern.

## 1. Introduction

The *bla*_OXA-48_ gene, encoding class D beta-lactamases, was first reported in *Klebsiella pneumoniae* from a patient with urinary tract and skin burns in Turkey in 2001 [1]. The enzyme usually hydrolyzed penicillins at high levels, but hydrolyzed carbapenems at a low level [2]. *Bla*_OXA-48_-like genes were widely reported among *K. pneumoniae* and other *Enterobacteriaceae* [3]. To date, 91 *bla*_OXA-48_-like variants have been identified, with classical *bla*_OXA-48_ being the most widespread [4,5,6,7]. *Bla*_OXA-48_-like gene carriers caused outbreaks of nosocomial and community infections in many countries, including China [3,8]. The number of reservoirs for these organisms was increasing among humans, animals and in the environment [3]. Thus, the rapid dissemination of carbapenem-resistant species harboring *bla*_OXA-48_-like genes in different ecosystems has posed a severe threat to human health.

*Shewanella xiamenensis*, Gram-negative rods, 0.7–0.8 × 2.5–4.0 μm, was isolated from coastal sediments for the first time in China [9]. *S. xiamenensis* was usually detected in the marine and freshwater environment, and rarely isolated from an animal source [10]. It has become an emerging pathogen contributing to intestinal colonization and abdominal cavity infection [11]. *S. xiamenensis* has been regarded as a progenitor of OXA-48 family class D beta-lactamase [12]. The chromosome-mediated *bla*_OXA-48_-like genes may be mobilized onto plasmids by insertion sequences, then plasmids could be transferred to clinically relevant pathogens (such as *Escherichia coli*). Increasing numbers of *bla*_OXA-48_-variants were reported in *S. xiamenensis* from human and environmental sources, such as *bla*_OXA-181_, *bla*_OXA-199_, *bla*_OXA-204_, and *bla*_OXA-538_ [13,14,15,16]. Thus, the purpose of this study was to characterize a gene belonging to the *bla*_OXA-48_ family, and investigate its genetic environment in *S. xiamenensis*.

## 2. Materials and Methods

### 2.1. Bacterial Isolate

In July 2015, 36 backyard farms were randomly selected in rural China and wastewater was sampled from the storage pool, which was located near the pig farms, using sterilized bottles. Water samples were filtered through 0.45 µm sterile membrane filters (Millipore, MA, USA), then membranes were inoculated in brain heart infusion broth (Oxoid, Basingstoke, UK) and cultured at 37 °C overnight. The enriched solutions (100 µL) were plated on MacConkey agar (Oxoid, Basingstoke, UK) with 2 mg/L meropenem (Meilun, Dalian, China) to isolate potential carbapenem-resistant isolates. The presence of the carbapenemase-encoding genes (*bla*_NDM-_, *bla*_KPC-_, *bla*_IMP-_ and *bla*_OXA-48_-types) in the potential isolates were tested by using PCR and sequencing (Biosune, Shanghai, China), as described previously [17]. Species confirmations were performed by matrix-assisted laser desorption ionization-time-of-flight mass spectrometry (bioMérieux, Lyon, France), sequencing of the 16S rRNA and gyrB genes.

### 2.2. Antibiotic Susceptibility Testing

The minimum inhibitory concentrations (MICs) of *S. xiamenensis* strain sx20 were determined using the broth microdilution method with the following agents: amikacin, ampicillin, ampicillin-sulbactam, aztreonam, cefazolin, ceftazidime, cefotetan, ceftriaxone, cefepime, ciprofloxacin, ertapenem, fosfomycin, gentamicin, imipenem, levofloxacin, meropenem, nitrofurantoin, piperacillin-tazobactam, tobramycin (Meilun, Dalian, China). The control strain was *E. coli* ATCC 25922. The results were interpreted according to EUCAST guidelines. Phenotypic detection of carbapenemase was performed using the Carba-direct NP test [18]. Bacteria were cultured on Mueller-Hinton agar overnight (Oxoid, Basingstoke, UK). The bacterial mass was scraped off with a 1-μL loop and suspended in a 1.5-mL Eppendorf tube containing 100 μL of 20 mM Tris-HCl lysis buffer. This lysate was mixed with 100 μL of an aqueous indicator solution which contained 0.05% phenol red with 0.1 mmol/L ZnSO_4_ and 6 mg/mL imipenem, and the phenol red solution without antibiotic as a control tube.

### 2.3. Whole-Genome Sequencing and Analysis

The entire genome of *S. xiamenensis* strain sx20 was sequenced using whole-genome sequencing (WGS) analysis using an Illumina HiSeq 4000-PE150 platform (Illumina, CA, USA). The sequences were assembled using SPAdes 3.11 and annotated via RAST (http://rast.nmpdr.org/). Antibiotic resistance genes and virulence genes were analyzed by Resfinder (https://cge.cbs.dtu.dk/services/ResFinder/) and the Virulence Factor Database (VFDB, http://www.mgc.ac.cn/VFs/). The genetic environment was visualized by Easyfig 2.2.3. Clustal Omega (https://www.ebi.ac.uk/Tools/msa/clustalo/) and Jalview [19] were used to perform the alignment analysis of amino acid sequences. The effect of the biological function of a protein caused by amino acid substitution or indel was predicted by PROVEAN (http://provean.jcvi.org/). The sequencing data of the whole genome and *bla*_OXA-894_ gene was deposited under the GenBank accession number SUNE00000000 and MN525568, respectively.

### 2.4. The Conjugation Assay

The conjugation experiment was carried out using the mixed broth method as previously described [20]. Conjugation was performed using *E. coli* J53 (sodium azide-resistant) as the recipient strain. Transconjugants were selected on LB agar plates (Oxoid, Basingstoke, UK) supplemented with sodium azide (100 mg/L) and meropenem (2 mg/L).

### 2.5. Phylogenetic Analysis of the Bla_OXA-48_-Like Genes

A phylogenetic tree of *bla*_OXA-48_-like genes was constructed by the MEGA X software [21] using the maximum likelihood method with 1000 bootstrapping. The representative sequences and closest references were collected from the GenBank database, including *bla*_OXA-10_ (NG_049393), *bla*_OXA-48_ (NG_049762), *bla*_OXA-48b_ (KC902850), *bla*_OXA-54_ (NG_049794), *bla*_OXA-162_ (NG_049461), *bla*_OXA-181_ (KX298210), *bla*_OXA-199_ (NG_049495), *bla*_OXA-204_ (KC902852), *bla*_OXA-244_ (NG_049539), *bla*_OXA-252_ (NG_050608), *bla*_OXA-416_ (KU198597), *bla*_OXA-515_ (NG_055476), *bla*_OXA-538_ (KX827284), *bla*_OXA-546_ (NG_054959), *bla*_OXA-894_ (MN525568) and *bla*_OXA-547_ (NG_054693).

## 3. Results and Discussion

In our study, *S. xiamenensis* strain sx20 was isolated from pig wastewater in rural China. It was resistant to ampicillin, aztreonam, ertapenem, imipenem, meropenem and fosfomycin. The Carba NP test showed it was a carbapenemase producer. Carbapenem MICs of *S. xiamenensis* strain sx20 were similar to *S. xiamenensis* IR34 harboring *bla*_OXA-204_ gene and *S. xiamenensis* DDP1 harboring *bla*_OXA-416_ (Table 1), but higher than strains of IR24 and IR33 harboring *bla*_OXA-48_ gene and S12-harboring *bla*_OXA-181_ gene [12,13,22], indicating there may be additional mechanisms for regulating carbapenem resistance in *S. xiamenensis.*

**Figure 1:**
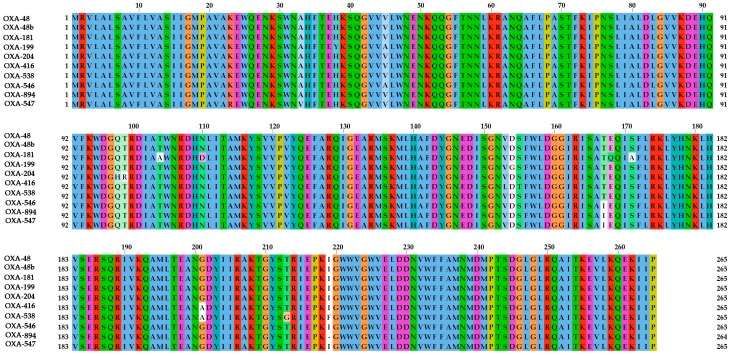
Alignment of the amino acid sequences of OXA-48 (WP_015059991), OXA-48b (AGS78031), OXA-181 (AQU42625), OXA-199 (WP_063861505), OXA-204 (AGS78037), OXA-416 (APO14326), OXA-538 (WP_071593227), OXA-546 (WP_087587945), OXA-894 (MN525568) and OXA-547 (WP_085562403).

One antibiotic resistance gene was identified in *S. xiamenensis* strain sx20. It is a new variant of the *bla*_OXA-546_ gene, namely *bla*_OXA-894_ (MN525568), which was 99.75% and 99.12% nucleotide identity to *bla*_OXA-546_ (KY682756) and *bla*_OXA-48_ (NG_049762). The OXA-894 differs from OXA-546 (A46V, I219*del*), OXA-48 (T167I, I219*del)* with two amino acid substitutions, respectively (Figure 1). The conjugation experiment was not successful in transferring *bla*_OXA-894_ gene to *E. coli* J53. The result of WGS confirmed that the *bla*_OXA-894_ gene was located on the chromosome. A number of chromosome-mediated *bla*_OXA-48_-like genes have been reported in *S. xiamenensis*, including *bla*_OXA-181_ gene [13], *bla*_OXA-199_ gene [15], *bla*_OXA-416_ gene [23] and *bla*_OXA-538_ gene [16]. Given this, our identification further supported the hypothesis that *S. xiamenensis* was the progenitor of *bla*_OXA-48_-like genes.

**Figure 2:**
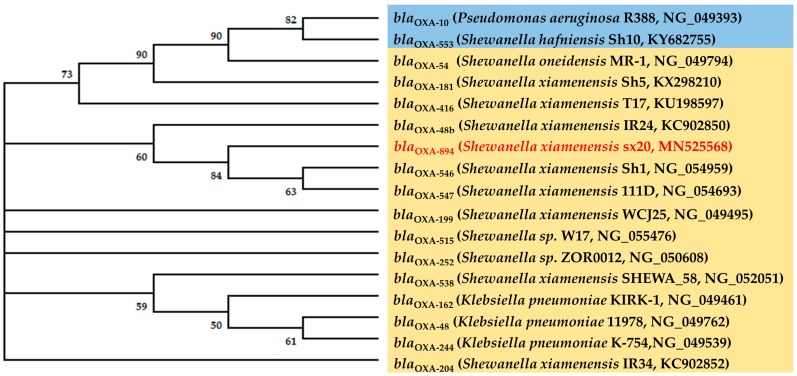
Molecular phylogenetic analysis using maximum likelihood method with 1000 bootstraps of *bla*_OXA-894_ with closest matches and representative nucleotide sequences retrieved from the GenBank database. The phylogenetic tree was constructed using MEGA X software. Bootstrap confidence is shown in %. The *bla*_OXA-48_-like genes are shown in yellow boxes, and other genes are highlighted in blue boxes.

The results of the phylogenetic analysis (Figure 2) showed that the *bla*_OXA-894_ gene formed a cluster with the *bla*_OXA-546_, *bla*_OXA-48b_, *bla*_OXA-547_ gene sequences detected in *S. xiamenensis*. The first such *bla*_OXA-48_-like gene in *S. xiamenensis* to be reported was from India, in 2011, namely *bla*_OXA-181_ gene, which has activity against carbapenems [13]. Until now, at least 10 *bla*_OXA-48_-like variants have been identified in the *S. xiamenensis* from India, China, Portugal, Italy and Algeria*,* including *bla*_OXA-48_, *bla*_OXA-48b_, *bla*_OXA-181_, *bla*_OXA-199_, *bla*_OXA-204_, *bla*_OXA-416_, *bla*_OXA-538_, *bla*_OXA-546_, *bla*_OXA-894_ and *bla*_OXA-547_ [10,12,13,14,15,16,22,23]. It indicated that the *bla*_OXA-48_-like genes are evolving continuously in different regions.

**Figure 3:**
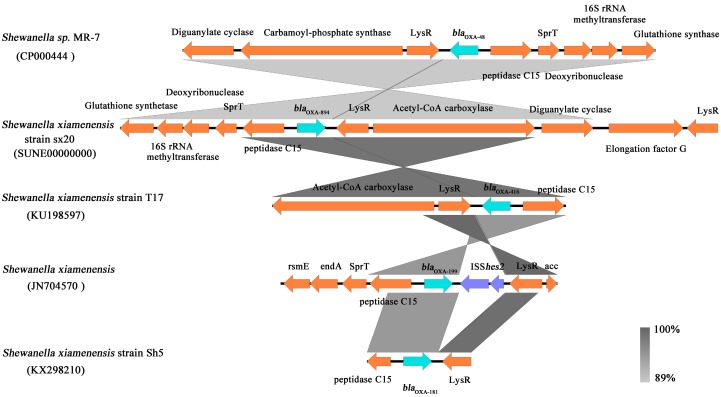
The genetic context of *bla*_OXA-48_-like gene in *S. xiamenensis* strain sx20 (SUNE00000000), *Shewanella spp.* MR-7 (CP000444), *S. xiamenensis* strain T17 (KU198597), *S. xiamenensis* strain WCJ25 (JN704570) and *S. xiamenensis* strain Sh5 (KX298210). Comparisons between multiple sequences were performed using Easyfig 2.2.3. Open reading frames are shown as arrows indicating the orientation of each gene. The gene name is shown over the corresponding arrow.

*Bla*_OXA-546_ was first reported in the plasmid of *S. xiamenensis* Sh1 isolated from saltmarsh plants in the USA, 2018 [14]. However, the chromosome-mediated *bla*_OXA-546_ gene has not been reported until now. In the current study, the single copy of the *bla*_OXA-894_ gene was found in *S. xiamenensis* sx20. It was located on the chromosome and inserted between the LysR family transcriptional regulator and the C15 gene (Figure 3). The genetic context of *bla*_OXA-894_ was similar to that previously reported for other *bla*_OXA-48_-like genes in the *Shewanella* species [14]. The occurrence of *bla*_OXA-894_ can increase the diversity of chromosome-mediated carbapenem-hydrolyzing class D β-lactamase genes in *Shewanella* species. No mobile element was found upstream and downstream of the *bla*_OXA-894_ gene in *S. xiamenensis* strain sx20, indicating a low probability of horizontal gene transfer. But *bla*_OXA-894_ gene was detected in an isolate from water, an environment that can be frequently affected by anthropogenic activities (such as discharges of wastewater), which may potentiate the spread of this gene in the environment, in animals and in humans. Therefore, the occurrence and potential health risk of carbapenem-resistant *S. xiamenensis* in water environment needs to be concerned.

According to the virulence factor database, only the *luxS* gene was detected with 82% nucleotide identity with wild type *luxS* (NC_000913). The deduced *LuxS* protein differs from wild-type *LuxS* (NP_417172) by 44 amino acid substitutions, and the T24H and T94V substitutions were predicted to be deleterious by PROVEAN. The *luxS* gene in the *Shewanella* encodes an autoinducer-2-like molecule which was the postulated universal bacterial signal. The mutants of the *luxS* gene could influence the biofilm formation, production of virulence factors and motility of pathogenic bacteria [24]. It indicated that the mutants of *luxS* gene may affect the virulence phenotype of the *S. xiamenensis* strain sx20.

## 4. Conclusions

This is the first report of chromosome-mediated *bla*_OXA-894_ gene in *S. xiamenensis.* The OXA-894 differs from OXA-546 (A46V, I219*del*), OXA-48 (T167I, I219*del*) with two amino acid substitutions, respectively. *Bla*_OXA-894_ gene was inserted between the LysR family transcriptional regulator and C15 gene. The occurrence of *bla*_OXA-894_ can increase the diversity of chromosome-encoded carbapenem-hydrolyzing class D β-lactamases identified in *Shewanella* species. A mutated *luxS* gene was also identified in this strain, which may affect the virulence phenotype of *S. xiamenensis*. The occurrence and potential health risk of carbapenem-resistant bacteria in the water environment is of concern.

## Figures and Tables

**Table 1:** The carbapenem MICs (mg/L) of *S. xiamenensis* harboring *bla*_OXA-48_-like genes.

Species	Year	*bla*_OXA-48_-Like Gene	Imipenem	Meropenem	Ertapenem	Reference
*S. xiamenensis* IR33	2013	*bla*_OXA-48_	4	1	8	[12]
*S. xiamenensis* Sh31	2018	*bla*_OXA-48_	8	2	8	[14]
*S. xiamenensis* IR24	2013	*bla*_OXA-48_-like	4	2	8	[12]
*S. xiamenensis* S12	2011	*bla*_OXA-181_	0.75	0.25	2	[13]
*S. xiamenensis* Sh5	2018	*bla*_OXA-181_	4	5	4	[14]
*S. xiamenensis* AS69	2017	*bla*_OXA-181_	0.75	-	-	[16]
*S. xiamenensis* AS85	2017	*bla*_OXA-181_	0.75	-	-	[16]
*S. xiamenensis* AS100	2017	*bla*_OXA-199_	0.5	-	-	[16]
*S. xiamenensis* IR34	2013	*bla*_OXA-204_	>32	8	>32	[12]
*S. xiamenensis* Sh33	2018	*bla*_OXA-204_	>32	8	>32	[14]
*S. xiamenensis* ZYW1	2019	*bla*_OXA-416_	1	-	-	[10]
*S. xiamenensis* DDP1	2013	*bla*_OXA-416_	32	16	>32	[22]
*S. xiamenensis* T17	2017	*bla*_OXA-416_	1	1	4	[23]
*S. xiamenensis* AS58	2017	*bla*_OXA-538_	3	-	-	[16]
*S. xiamenensis* Sh1	2018	*bla*_OXA-546_	1	0.5	3	[14]
*S. xiamenensis* sx20	2019	*bla*_OXA-894_	32	8	8	This study

Year means the publication time of the strain.
